# Tumor Heterogeneity in Breast Cancer

**DOI:** 10.3389/fmed.2017.00227

**Published:** 2017-12-08

**Authors:** Gulisa Turashvili, Edi Brogi

**Affiliations:** ^1^Department of Pathology, Memorial Sloan Kettering Cancer Center, New York, NY, United States

**Keywords:** breast cancer, tumor heterogeneity, histopathology, biomarkers, genetic markers

## Abstract

Breast cancer is a heterogeneous disease and differs greatly among different patients (intertumor heterogeneity) and even within each individual tumor (intratumor heterogeneity). Clinical and morphologic intertumor heterogeneity is reflected by staging systems and histopathologic classification of breast cancer. Heterogeneity in the expression of established prognostic and predictive biomarkers, hormone receptors, and human epidermal growth factor receptor 2 oncoprotein is the basis for targeted treatment. Molecular classifications are indicators of genetic tumor heterogeneity, which is probed with multigene assays and can lead to improved stratification into low- and high-risk groups for personalized therapy. Intratumor heterogeneity occurs at the morphologic, genomic, transcriptomic, and proteomic levels, creating diagnostic and therapeutic challenges. Understanding the molecular and cellular mechanisms of tumor heterogeneity that are relevant to the development of treatment resistance is a major area of research. Despite the improved knowledge of the complex genetic and phenotypic features underpinning tumor heterogeneity, there has been only limited advancement in diagnostic, prognostic, or predictive strategies for breast cancer. The current guidelines for reporting of biomarkers aim to maximize patient eligibility for targeted therapy, but do not take into account intratumor heterogeneity. The molecular classification of breast cancer is not implemented in routine clinical practice. Additional studies and in-depth analysis are required to understand the clinical significance of rapidly accumulating data. This review highlights inter- and intratumor heterogeneity of breast carcinoma with special emphasis on pathologic findings, and provides insights into the clinical significance of molecular and cellular mechanisms of heterogeneity.

## Introduction

Tumor heterogeneity is one of the hallmarks of malignancy. Intertumor heterogeneity is observed in breast carcinomas from different individuals. Intratumor heterogeneity is due to the presence of heterogeneous cell populations within an individual tumor (1). Early reports defined tumor heterogeneity based on the identification of intratumor cell populations with different characteristics, including tumorigenicity, treatment resistance, and metastatic potential (2–4). Although the heterogeneity of breast cancer at the cellular level was recognized already in the nineteenth century (5), its clinical relevance was first established about 30 years ago, with the introduction of estrogen receptor (ER) testing (6). Variation in the expression of ER among different tumors or distinct cell populations within a single tumor was thought to account for differences in clinical behavior and treatment response (6). Currently, understanding the molecular and cellular mechanisms of tumor heterogeneity that are relevant to the diagnosis, prognosis, and therapy of breast cancer is subject of intense research.

## Intertumor Heterogeneity

### Clinical and Histopathologic Heterogeneity

Intertumor heterogeneity of breast cancer is best illustrated by clinical staging of the disease based on physical examination and imaging findings. The TNM staging system by the American Joint Committee on Cancer (AJCC)/Union for International Cancer Control (UICC) incorporates Tumor size, regional lymph Node status, and distant Metastases (7). Standard breast cancer treatment is based on the tumor characteristics, including clinical stage, histopathologic features, and biomarker profile, and is affected by the patient’s age, menopausal status, and general health (8). The aforementioned traditional clinicopathologic variables have a profound impact on survival, and account for most of the differences in clinical outcome among patients with breast cancer (9).

The morphologic heterogeneity of breast carcinoma constitutes the basis for the histopathologic classification of breast cancer. Invasive ductal carcinoma (IDC) of no special type or not otherwise specified (NOS) is the most common (40–75%) histologic type of invasive breast cancer. Albeit common, IDC NOS is not at all well defined, and the 2012 World Health Organization (WHO) classification defines IDC NOS by exclusion, as “the heterogeneous group of tumors that fail to exhibit sufficient characteristics to achieve classification as a specific histological type” (9). In addition to IDC NOS, the WHO classification includes 21 special subtypes with distinctive morphologic features, of which invasive lobular carcinoma (ILC) is the most frequent (5–15%) (9). The other special subtypes of breast carcinoma are rare and differ significantly with regard to prognosis and response to adjuvant treatment (10–13). Tubular, mucinous, and papillary carcinomas usually have excellent clinical outcome compared to IDC and ILC (14, 15) and are not always treated with chemotherapy (16). By contrast, metaplastic carcinoma and poorly differentiated IDC NOS have a significantly worse outcome and are routinely treated with systemic chemotherapy (9).

The grade of breast carcinoma also highlights its tumor heterogeneity. Grade is assessed according to a 3-tier (low, intermediate, high) system based on the evaluation of three morphologic parameters, namely the percentage of the tumor arranged in glands and tubular structures, the degree of nuclear pleomorphism, and the mitotic rate (17). The grade of breast carcinoma is a robust prognostic factor, and is incorporated in clinical decision-making tools, such as the Nottingham Prognostic Index and Adjuvant! Online (9, 18). Breast cancers of different grades also show different profiles by proteomic, genomic and transcriptomic analysis (19–21). In multivariate models that include gene signatures, grade remains an independent prognostic factor for ER-positive tumors (22). Grade 1 and 3 breast carcinomas likely represent two very different diseases, and molecular data indicate that the progression from low- to high-grade carcinoma is exceedingly rare (9).

### Biomarker Heterogeneity

The expression of ER, progesterone receptor (PR), and human epidermal growth factor receptor 2 (HER2) is assessed routinely in all invasive breast carcinomas by immunohistochemistry (IHC) according to the recommendations by American Society of Clinical Oncology/College of American Pathologist (ASCO/CAP) (23, 24). The aforementioned biomarkers are established prognostic and predictive factors and their expression in breast carcinomas is critical in guiding patient treatment (8, 25).

Estrogen receptor and PR are expressed in approximately 80% and 60–70% of breast carcinomas, respectively (26, 27). Although ER-positive tumors co-express PR (ER+/PR+) in 70–80% of cases, some breast carcinomas are ER+/PR− or, rarely, ER−/PR+. The response to hormonal treatment also varies, with the best response (approximate rate of 60%) in ER+/PR+ tumors and lower rates in ER+/PR− and ER−/PR+ tumors (9).

The HER2 oncoprotein is overexpressed in approximately 15–20% of primary breast carcinoma as detected by IHC staining using the approved reagents, testing protocols, and scoring algorithm. Positive (3+) HER2 staining highly correlates with gene amplification (9); depending on the definition of HER2-equivocal (2+) staining, approximately 10–20% of HER2-equivocal breast carcinomas are found to be HER2-amplified by *in situ* hybridization (ISH). HER2-positive breast carcinomas have the most unfavorable prognosis of all types of invasive breast cancers, but they show high rate of response to anti-HER2 targeted therapy (e.g., trastuzumab, lapatinib) (28), as documented by the pathologic complete response post-neoadjuvant treatment in about 50–60% of patients with HER2-positive tumors (29).

Breast carcinomas that do not express ER, PR, and HER2, usually referred to as “triple-negative” breast carcinomas, constitute an extremely heterogeneous group histologically, genetically, prognostically as well as with regard to treatment response. Emerging data suggest that nuclear expression of the androgen receptor (AR) can be detected in 12–55% of triple-negative (ER-/PR-/HER2-) breast cancer (30–32). The prognostic significance of AR expression in triple-negative carcinomas is controversial, but it is associated with improved survival in other tumor subtypes (33). Ongoing clinical trials evaluating AR antagonists (such as bicalutamide and enzalutamide) in AR+ (defined as nuclear staining in ≥10% of tumor cells by IHC) triple-negative breast carcinomas show promising results (31, 34). AR positivity is associated with lower Ki-67 proliferation index, suggesting that AR may promote a stem-like or mesenchymal phenotype in this subset of tumors (32). No standardized assays or guidelines for evaluating the AR expression in breast carcinoma are available at present.

Hundreds of other biomarkers have been investigated in breast cancer for potential diagnostic, prognostic, and therapeutic implications. Functional classification of these biomarkers includes growth and proliferation (Ki-67, survivin, NGAL), invasion and metastasis (p53, MMP-9, SK1, DcR3, COX2, EZH2, microRNAs miR-105, and miR126), epithelial–mesenchymal transition (EMT) (WNT5A/B, Pea3), immune response (PD-L1), therapy resistance (HER2Δ16, pSTS3, KLK10), survival (miR-574-3p, miR-660-5p, PIWIL3, PIWIL4), and many others (35). The magnitude of the effect of tumor heterogeneity on biomarker expression or its clinical significance remains uncertain. A systematic approach and standardized quantitative reporting of biomarkers is required to better guide therapeutic decisions.

### Genetic Heterogeneity

Gene expression analysis classifies breast cancer into four major intrinsic molecular subtypes with prognostic and therapy implications: luminal A, luminal B, HER2-enriched, and basal-like (36). The luminal A and luminal B subtypes exemplify tumor heterogeneity within ER-positive breast carcinomas and have better survival than HER2-enriched and basal-like subtypes. Both luminal subtypes express ER, but the luminal B tumors are characterized by increased expression of proliferation-associated genes and have worse prognosis than luminal A tumors (37). The HER2-enriched subtype is characterized by increased expression of HER2 and proliferation genes and includes ER-/PR-/HER2+ and ER+/PR+/HER2+ tumors. The basal-like subtype is enriched for genes expressed in basal epithelial cells, and is triple-negative in 70% of cases (36). Additional subtypes include claudin-low tumors with stem-like signature (38) and AR-positive molecular apocrine tumors (39). Meta-analysis of gene expression studies suggests that the prognostic impact of different signatures is related to the proliferation-associated genes (40). Although gene expression profiles can predict response to chemotherapy and recurrence risk (41), classification of breast carcinoma based on gene expression is hindered by clinical and molecular heterogeneity. Patients with breast carcinoma of the same molecular subtype and receiving identical treatments may have different clinical outcomes and/or acquire resistance to therapy (42). Frequent (>10%) somatic mutations in TP53, PIK3CA, and GATA3 have been documented in breast carcinomas (43). More recent studies have yielded other molecular subgroups, including a molecular classification based on integrated genomic and transcriptomic profiling of 2,000 breast tumors yielding 10 novel subtypes of breast cancer with distinct clinical outcomes (44, 45). Additional studies are needed to evaluate the practical clinical relevance and treatment implications of driver-based breast cancer classifications.

RNA-based multigene expression assays have been developed to estimate recurrence risk in ER-positive and/or lymph node-negative patients. According to the ASCO clinical practice guidelines (8), some multigene expression assays show sufficient evidence for clinical utility. They include the 21-gene assay Oncotype DX (46), the 11-gene EndoPredict (47), the 50-gene assay Prosigna based on the prediction analysis of microarray 50 model (48–50), and the 7-gene based Breast Cancer Index (BCI) (51). Prosigna, BCI, and EndoPredict predict late recurrence and subclassify tumors into molecular subtypes (52). Oncotype Dx is a reverse transcriptase polymerase chain reaction-based assay, and quantifies the likelihood of early distant recurrence and chemotherapy benefit for patients with lymph node-negative, hormone receptor-positive, HER2-negative breast cancer (46, 53). The risk of recurrence is expressed as a numerical value between 0 and 100, referred to as recurrence score (RS). Tumors are stratified into low risk (RS ≤ 17), intermediate risk (RS 18–30), and high risk (RS ≥ 31) categories (46). In patients with tumors of RS ≤ 17, the benefit of chemotherapy is quantified as too small (2%) to outweigh its possible side effects. By contrast, patients with RS ≥ 31 greatly benefit from chemotherapy due to their increased (28%) recurrence risk (54). The clinical management of intermediate risk patients is more varied and includes endocrine therapy with or without chemotherapy, depending on the patient’s clinicopathologic characteristics and individual preference. Two ongoing clinical trials aim to further stratify the benefit of chemotherapy in patients with intermediate RS who are clinically node-negative (TailorX) or node-positive (RxSponder) at presentation.

Due to the costs, time and technical expertise required for molecular assays, IHC stains have been evaluated as possible alternative methods for indirect assessment of molecular subtype that can be used in most laboratories. The IHC staining panel comprising ER, PR, HER2, Ki-67, epidermal growth factor receptor (EGFR) and cytokeratin 5/6 (CK5/6) can identify the molecular subtypes of breast cancer with satisfactory and reproducible accuracy: (1) Luminal A (ER+/PR±/HER2-/Ki-67−); (2) Luminal B (ER+/PR±/HER2−/Ki-67+; with Ki-67-positivity defined as ≥14%); (3) Luminal/HER2+ (HER2+/ER+/PR ±); (4) HER2+ (HER2+/ER−/PR−); and (5) Basal, including core basal (ER−/PR−/HER2−/EGFR+ or CK5/6+), and five-marker negative (ER−/PR−/HER2−/EGFR−/CK5/6−) subgroups (55, 56). Considering that not all triple-negative tumors are basal-like and *vice versa*, and that ER-positive luminal tumors are highly diverse, genetic heterogeneity of breast cancer is likely far more complex than our current understanding of this multidimensional issue or the existing molecular classifications. Development of assays integrating multigene tests with mutational or genomic profiles is required to better elucidate the interplay and clinical significance of prognostic and predictive molecular drivers in ER-positive breast cancer (52).

## Intratumor Heterogeneity

### Histopathologic Heterogeneity

**Figure 1:**
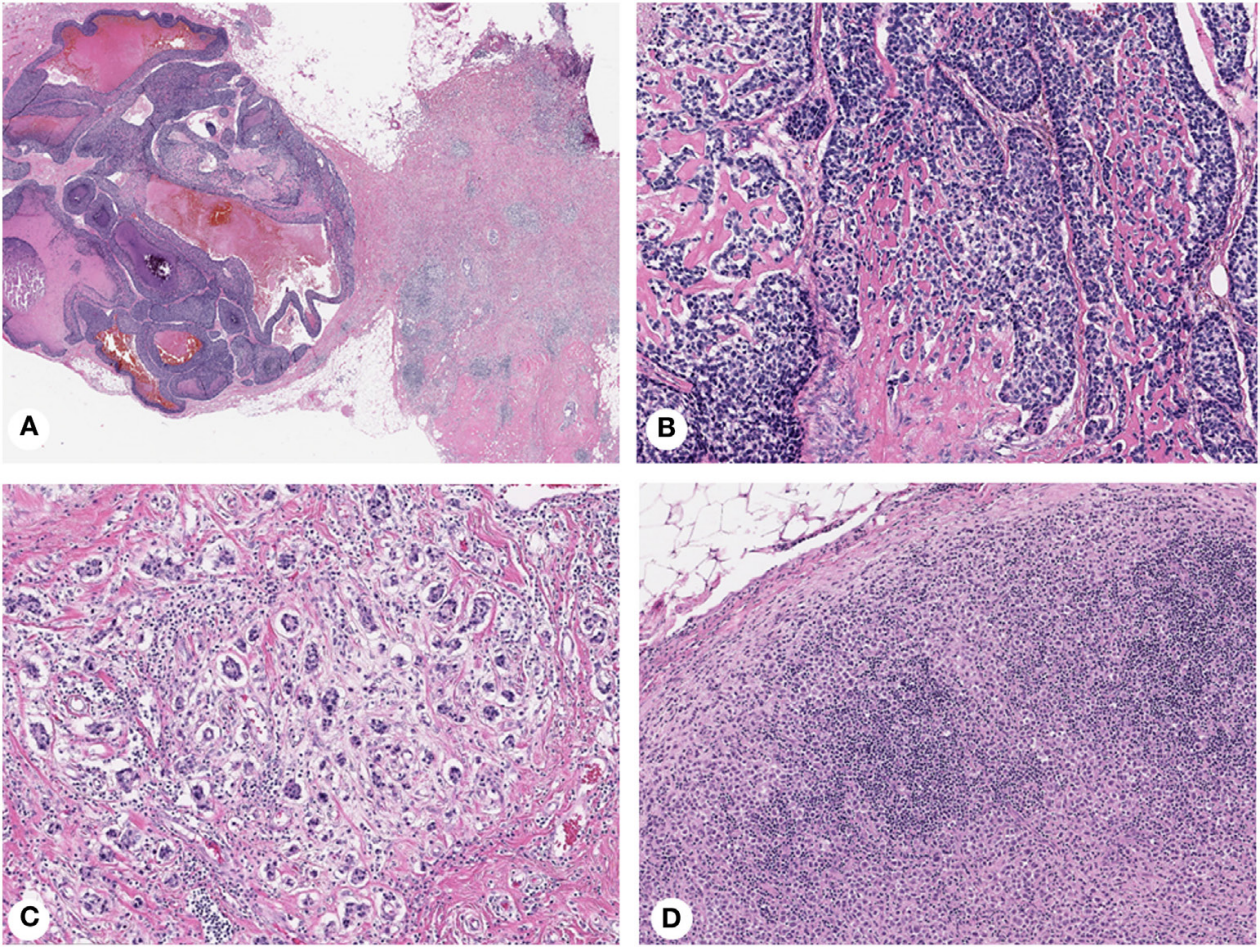
Histopathologic heterogeneity of breast cancer: invasive mammary carcinoma with mixed morphology **(A)**, composed of basaloid areas with osteoid production **(B)** and ductal not otherwise specified **(C)** components. Lymph node metastasis showing a diffuse pattern of tumor growth **(D)**. Magnification: 100× **(A)**, 200× **(B–D)**; Hematoxylin-eosin staining.

Morphologic intratumor heterogeneity can be appreciated as variability in different areas of tumor (spatial heterogeneity), or as tumor progression over time (temporal heterogeneity) (1). Spatial heterogeneity is readily appreciated in daily surgical pathology practice within a single tumor, but can also be detected between primary breast carcinoma and synchronous lymph node metastases, and even between synchronous metastases from different sites. Breast carcinomas with truly mixed morphology consist of two morphologically different components (e.g., IDC and mucinous carcinoma), but other tumors exhibit ambiguous morphologic features (e.g., IDC with lobular features) or contain foci of distinct differentiation (e.g., IDC with focal squamous/basaloid or spindle cell differentiation) (Figure 1). Morphologically distinct areas within individual tumors can be clonal with specific genetic aberrations (57–59). Temporal heterogeneity includes evolution of an invasive tumor over time or in response to therapy (60, 61), development of asynchronous metastatic disease (62, 63) and progression from *in situ* to invasive carcinoma (64, 65).

Although current clinical management of breast cancer is guided by histologic, IHC, and molecular characteristics of the primary tumor, treatment efficacy may be affected by altered morphologic and IHC features in metastases (1, 66). Discordance rates include 16–33.6% for ER, 32–40% for PR, and 10–15.7% for HER2 (67–69). Furthermore, women with discordant ER-staining results between primary and metastatic breast carcinoma had a 48% increased risk of death in one study (69). Variability in biomarker expression between primary and metastatic tumors can be due to treatment (70) or may occur in the absence of therapeutic intervention (67, 69, 71, 72). Significant variations have also been reported in genomic heterogeneity (62, 73), single nucleotide or copy number variants (63, 66, 74, 75), and chromosomal rearrangements and insertion/deletions (75). Due to insufficient evidence that changing treatment based on the altered biomarker status affects patient outcome, the current practice guidelines only recommend biopsying and retesting ER/PR/HER2 on accessible metastases if clinically indicated (76).

### Biomarker Heterogeneity

**Figure 2:**
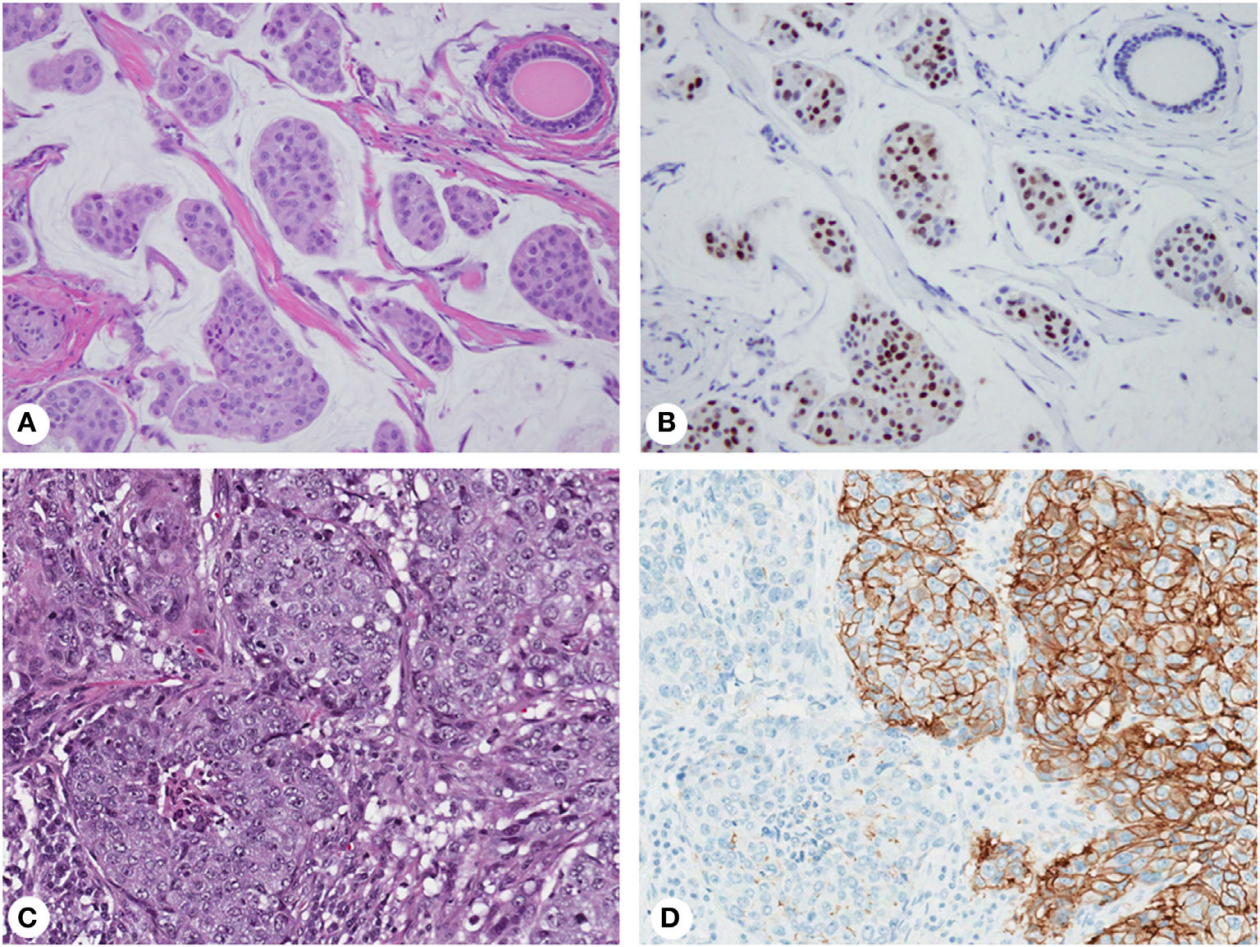
Biomarker heterogeneity of breast cancer: mucinous carcinoma **(A)** with variable expression of estrogen receptor from no immunoreactivity to nuclear staining with weak to strong intensity **(B)**; invasive ductal carcinoma **(C)** with areas of 3+ (positive) and 1+ (negative) membranous staining for human epidermal growth factor receptor 2 **(D)**. Magnification: 200× **(A–D)**; hematoxylin-eosin staining **(A,C)** and immunohistochemistry **(B,D)**.

Expression of biomarkers can be highly variable within an individual tumor (Figure 2) causing interpretation problems and discordant results in small biopsies. ER/PR staining variations within a single tumor have long been recognized (77, 78). The proportion of ER/PR-expressing tumor cells in individual tumors varies from 1 to 100%, and expression levels directly correlate with response to endocrine therapy (26, 27). However, even tumors with very low levels (1% of tumor cells) may respond, justifying the use of the 1% cutoff for ER/PR-positivity by the ASCO/CAP guidelines (23). Nevertheless, this approach does not consider intratumor heterogeneity, accounting for limited clinical significance of classifying tumors with unequal distribution of ER-expressing cells as ER-positive (52).

Human epidermal growth factor receptor 2 (HER2) IHC staining and gene amplification can be highly heterogeneous (78–80) and affect disease-free survival (81). Discrepant HER2 IHC results ranges from 1 to >50% (79, 82, 83), while the rate of gene amplification heterogeneity is 5–30% (84). By IHC, HER2-positive tumors show complete, intense, circumferential membrane staining in 10–100% of tumor cells (3+ staining). Some tumors exhibit incomplete and/or weak-to-moderate circumferential membrane staining in >10% of cells or complete, intense, circumferential membrane staining in ≤10% of cells (2+ staining) by IHC but gene amplification by ISH (24). Some cases have protein overexpression without gene amplification, amplification without protein overexpression, or marked intratumor heterogeneity. Although the ASCO/CAP guidelines acknowledge heterogeneous amplification and recommend reporting separate areas (84), detecting gene amplification in one area is sufficient to consider a tumor HER2-amplified. This approach maximizes patient eligibility for targeted therapy without considering clinical implications of intratumor heterogeneity (52).

Other biomarkers with heterogeneous expression include EGFR (85), p53 (78, 85), c-myc (82), and proliferation markers, including Ki-67 (78, 85, 86), cyclin-D1 (82), and PCNA (87). Ki-67 is a non-histone nuclear protein expressed in all phases of the cell cycle except G0. It has been shown to have a prognostic and predictive value in both ER-positive and ER-negative breast carcinomas (88–90). However, expression levels of Ki-67 can be notoriously higher at the tumor periphery with variable staining throughout the tumor in the form of hot spots (91). Furthermore, intratumor heterogeneity of Ki-67 expression can occur in breast carcinomas of various histologic subtypes and grades (86). Several scoring systems have been suggested for the assessment of Ki-67 staining, including evaluating the hot spots alone, calculating the average score including hot spots, or even avoiding them altogether (91). In contrast to primary tumors, lymph node metastases have been reported to have a homogeneous distribution of Ki-67 expression. Moreover, metastatic tumor cells were highly proliferative and associated with Ki-67 levels in the highest expression hot spots in primary tumors. This may reflect the temporal heterogeneity through clonal expansion of the primary tumor growth fraction with metastatic potential (92).

It is unclear whether intratumor heterogeneity represents a true biologic phenomenon or a technical artifact due to poor fixation and/or processing (78, 93). Nonetheless, extensive sampling and IHC testing with adequate negative and positive controls are always prudent.

### Circulating Tumor Cells (CTCs)

Circulating tumor cells are cancer cells that detach from a primary tumor and circulate in the bloodstream during cancer progression (94). CTCs have been reported in 26% of metastatic breast tumors (95). CTC count is an independent predictor of poor survival, treatment resistance, and early recurrence in some studies (96–104). However, practical application of CTC-based assays as “liquid biopsies” is limited by significant molecular and functional heterogeneity of CTCs (105–107), including variability at the protein (HER2, ER, Ki-67) (76, 108–114) and gene (PIK3CA) levels (76, 108–114), and EMT (115, 116). During the process of EMT, which is thought to precede the development of lymphovascular invasion and metastasis, the tumor cells lose epithelial characteristics, such as cell polarity, cell-to-cell adhesion, and expression of epithelial markers (EpCAM), and acquire mesenchymal properties including motility and invasiveness (115, 116). The presence of EMT in CTCs indicates a poor prognosis (117). Discordant HER2-expression in CTCs in particularly relevant (118–121), and clinical trials (DETECT, TREAT-CTC) are underway to evaluate treatment options based on the HER2 status of CTCs in metastatic breast cancer (122, 123). Heterogeneity in CTCs is thought to represent one of the mechanisms of resistance to endocrine therapy (1). Nonetheless, due to insufficient clinical evidence, the ASCO guidelines do not recommend changing therapy solely on the basis of CTC counts for monitoring treatment response (76).

### Genetic Heterogeneity

Breast cancer shows considerable intratumor heterogeneity with regard to chromosomal and genomic alterations (44, 124–130) which affect many processes and functions, such as signaling pathways, antitumor immunity, cell senescence, migration and metastasis, angiogenesis, treatment response, and metabolic pathways (52). Different cell clones can either segregate in different areas of the tumor or scatter and intermingle within the same area (131). Complexity of intratumor genetic heterogeneity is best exemplified by a study of 100 tumors which identified driver mutations in >40 cancer genes, including AKT2, ARID1B, CASP8, CDKN1B, MAP3K1, MAP3K13, NCOR1, SMARCD1 and TBX3, and 73 combinations of mutated genes (129). Intratumor genetic heterogeneity can be characterized by bulk sequencing and single-cell or single-molecule sequencing (132). Bulk tumor sequencing cannot determine the cellular origin of molecular changes, location within tumors or the degree of heterogeneity, while single-cell sequencing cannot provide information on the remaining cell population, limiting their clinical use in clinical practice (52). An autopsy study comparing the molecular alterations in multiple synchronous metastases of breast carcinoma documented molecular evolution and clone selection of tumor cells in response to targeted treatment, and highlighted the challenges to targeted treatment posed by the complex molecular heterogeneity of metastatic disease (133).

### Non-Genetic (Epigenetic) Heterogeneity

Epigenetic heterogeneity is defined as modifications in gene expression without DNA sequence changes (52, 134, 135). In breast cancer, epigenetic silencing through histone modification or DNA methylation can affect tumor suppressor genes including p16INK4A (136) and RASSF1A (137), and ER/PR/HER2 (138). Transient phenotypic variants of cells can also arise due to stochastic changes in the biochemical processes within cells (135), which might involve changes in chromatin states or mRNAs (139) and affect sensitivity to therapy (139). The clinical significance of non-genetic heterogeneity remains to be determined.

## Four Mechanisms of Breast Cancer Heterogeneity

### Differentiation State of the Cell-Of-Origin

Each mammary cell type has a specific molecular profile (140, 141). Tumor phenotype is determined by the combination of this differentiation state and the tumor-initiating genetic event. Distinct differentiation states of human mammary epithelial cells grown in cell cultures lead to different tumor subtypes in mouse xenografts (142, 143), e.g., EpCAM+ cells form epithelial tumors with variable ER-positivity, while CD10+ cells are precursors of metaplastic carcinoma (144). Multiple phenotypes can arise from one cell-of-origin depending on the initiating genetic event, e.g., HER2-expression in luminal cells forms luminal tumors, while BRCA1/2 leads to basal differentiation (145, 146). Furthermore, expression of the same oncogene (e.g., PIK3CA) in luminal cells can lead to different tumor types (147), while BRCA2/TP53 depletion results in IDC and metaplastic carcinoma in luminal cells, but myoepithelial carcinoma in basal cells (141). Nevertheless, the final tumor phenotype does not always reflect the cell-of-origin (141).

### Cell Plasticity

The equilibrium of cell states within tumors is maintained by dynamic bidirectional cell conversions between “cancer stem cells” (CSCs) and non-CSCs (148). CSCs self-renew and form more stem cells, differentiated cells, and tumor cells (149), while differentiated tumor cells can dedifferentiate (150). Cell plasticity may involve EMT and PIK3CA-expression (147, 151, 152).

### Genetic Evolution of Cancer

Tumorigenesis is a multi-step evolutionary process driven by Darwinian selection of the fittest cells and genetic instability (149, 153). Although most tumors arise from a single cell due to the initiating genetic event (“driver mutation”), cancer cells acquire additional aberrations during tumor evolution and, thus, each tumor contains multiple subclones harboring “passenger mutations” (132). Cell plasticity and genetic evolution may overlap as CSCs evolve and change in frequency due to clonal evolution during tumor progression (149).

### Tumor Microenvironment

Tumor stroma contains fibroblasts, blood vessels, and immunocompetent cells. Interactions between this non-cancerous microenvironment and tumor cells can contribute to carcinogenesis (154), exemplified by decreased sensitivity of tumor cells to growth inhibitors (155) and suppressed tumor growth by microvasculature (156).

### Clinical Implications

Despite our improved understanding of complex phenotypic and genetic aspects of tumor heterogeneity, no significant clinical progress has been made with regards to incorporating this knowledge into effective diagnostic, prognostic, and therapeutic strategies for breast cancer (52). Patients are managed based on the ER/PR/HER2 status of the primary tumor, and metastatic sites may not always biopsied for histologic confirmation or biomarker retesting (68). Since “actionable” mutations in the initial tumor may no longer be responsible for tumor progression, it is essential to identify the dominant clones driving metastatic disease and treatment resistance (157, 158). Ideally, intratumor heterogeneity should be assessed by sequencing technologies at diagnosis for each patient, followed by monitoring of clonal dynamics during disease progression and treatment. This will allow for the identification of genetic changes driving resistance as well as therapy adjustments (1, 141, 159). Potential strategies to overcome treatment resistance include targeting driver mutations and deleterious passenger mutations, and modulating the tumor microenvironment and immunotherapy (93). Further well-designed studies are required to elucidate the clinical validity of rapidly accumulating data.

## Author Contributions

GT: writing original draft and editing. EB: writing, reviewing, and editing.

## Conflict of Interest Statement

The authors declare that the research was conducted in the absence of any commercial or financial relationships that could be construed as a potential conflict of interest.

## References

[1] Ellsworth RE Blackburn HL Shriver CD Soon-Shiong P Ellsworth DL Molecular heterogeneity in breast cancer: state of the science and implications for patient care. Semin Cell Dev Biol (2016) 64:65–72. 10.1016/j.semcdb.2016.08.025 27569190

[2] Fidler IJ Kripke ML. Metastasis results from preexisting variant cells within a malignant tumor. Science (1977) 197:893–5. 10.1126/science.887927 887927

[3] Fidler IJ. Tumor heterogeneity and the biology of cancer invasion and metastasis. Cancer Res (1978) 38:2651–60. 354778

[4] Miller FR Miller BE Heppner GH. Characterization of metastatic heterogeneity among subpopulations of a single mouse mammary tumor: heterogeneity in phenotypic stability. Invasion Metastasis (1983) 3:22–31. 6677618

[5] Young RH Louis DN. The Warrens and other pioneering clinician pathologists of the Massachusetts General Hospital during its early years: an appreciation on the 200th anniversary of the hospital founding. Mod Pathol (2011) 24:1285–94. 10.1038/modpathol.2011.132 21926958

[6] Hawkins RA Killen E Tesdale AL Sangster K Thomson M Steele RJ Oestrogen receptors, lactate dehydrogenase and cellularity in human breast cancer. Clin Chim Acta (1988) 175:89–96. 10.1016/0009-8981(88)90038-1 3168286

[7] Hortobagyi GN D’orsi CJ Edge SB Mittendorf EA Rugo HS Solin LJ AJCC Cancer Staging Manual – Breast. 8th ed Chicago: Springer (2017).

[8] Harris LN Ismaila N Mcshane LM Andre F Collyar DE Gonzalez-Angulo AM Use of biomarkers to guide decisions on adjuvant systemic therapy for women with early-stage invasive breast cancer: American Society of Clinical Oncology Clinical Practice Guideline. J Clin Oncol (2016) 34:1134–50. 10.1200/JCO.2015.65.2289 26858339 PMC4933134

[9] Lakhani SR Ellis IO Schnitt SJ Tan PH Van De Vijver MJ, editors. WHO Classification of Tumours of the Breast. Lyon: France International Agency for Research on Cancer (2012).

[10] Page DL. Special types of invasive breast cancer, with clinical implications. Am J Surg Pathol (2003) 27:832–5. 10.1097/00000478-200306000-00016 12766589

[11] Yerushalmi R Hayes MM Gelmon KA Breast carcinoma – rare types: review of the literature. Ann Oncol (2009) 20:1763–70. 10.1093/annonc/mdp245 19602565

[12] Weigelt B Geyer FC Reis-Filho JS. Histological types of breast cancer: how special are they? Mol Oncol (2010) 4:192–208. 10.1016/j.molonc.2010.04.004 20452298 PMC5527938

[13] Nagao T Kinoshita T Hojo T Tsuda H Tamura K Fujiwara Y. The differences in the histological types of breast cancer and the response to neoadjuvant chemotherapy: the relationship between the outcome and the clinicopathological characteristics. Breast (2012) 21:289–95. 10.1016/j.breast.2011.12.011 22277312

[14] Rosen PP Groshen S Kinne DW Norton L. Factors influencing prognosis in node-negative breast carcinoma: analysis of 767 T1N0M0/T2N0M0 patients with long-term follow-up. J Clin Oncol (1993) 11:2090–100. 10.1200/JCO.1993.11.11.2090 8229123

[15] Rakha EA Lee AH Evans AJ Menon S Assad NY Hodi Z Tubular carcinoma of the breast: further evidence to support its excellent prognosis. J Clin Oncol (2010) 28:99–104. 10.1200/JCO.2009.23.5051 19917872

[16] Colleoni M Russo L Dellapasqua S. Adjuvant therapies for special types of breast cancer. Breast (2011) 20(Suppl 3):S153–7. 10.1016/S0960-9776(11)70315-0 22015285

[17] Elston CW Ellis IO. Pathological prognostic factors in breast cancer. I. The value of histological grade in breast cancer: experience from a large study with long-term follow-up. Histopathology (1991) 19:403–10. 10.1111/j.1365-2559.1991.tb00229.x 1757079

[18] Rakha EA Reis-Filho JS Baehner F Dabbs DJ Decker T Eusebi V Breast cancer prognostic classification in the molecular era: the role of histological grade. Breast Cancer Res (2010) 12:207 10.1186/bcr2607 20804570 PMC2949637

[19] Abd El-Rehim DM Ball G Pinder SE Rakha E Paish C Robertson JF High-throughput protein expression analysis using tissue microarray technology of a large well-characterised series identifies biologically distinct classes of breast cancer confirming recent cDNA expression analyses. Int J Cancer (2005) 116:340–50. 10.1002/ijc.21004 15818618

[20] Sotiriou C Wirapati P Loi S Harris A Fox S Smeds J Gene expression profiling in breast cancer: understanding the molecular basis of histologic grade to improve prognosis. J Natl Cancer Inst (2006) 98:262–72. 10.1093/jnci/djj052 16478745

[21] Natrajan R Lambros MB Rodriguez-Pinilla SM Moreno-Bueno G Tan DS Marchio C Tiling path genomic profiling of grade 3 invasive ductal breast cancers. Clin Cancer Res (2009) 15:2711–22. 10.1158/1078-0432.CCR-08-1878 19318498

[22] Yu K Lee CH Tan PH Hong GS Wee SB Wong CY A molecular signature of the Nottingham prognostic index in breast cancer. Cancer Res (2004) 64:2962–8. 10.1158/0008-5472.CAN-03-2430 15126326

[23] Hammond ME Hayes DF Dowsett M Allred DC Hagerty KL Badve S American Society of Clinical Oncology/College of American Pathologists Guideline recommendations for immunohistochemical testing of estrogen and progesterone receptors in breast cancer. J Clin Oncol (2010) 28:2784–95. 10.1200/JCO.2009.25.6529 20404251 PMC2881855

[24] Wolff AC Hammond ME Hicks DG Dowsett M Mcshane LM Allison KH Recommendations for human epidermal growth factor receptor 2 testing in breast cancer: American Society of Clinical Oncology/College of American Pathologists clinical practice guideline update. J Clin Oncol (2013) 31:3997–4013. 10.1200/JCO.2013.50.9984 24101045

[25] Early Breast Cancer Trialists’ Collaborative Group. Effects of chemotherapy and hormonal therapy for early breast cancer on recurrence and 15-year survival: an overview of the randomised trials. Lancet (2005) 365:1687–717. 10.1016/S0140-6736(05)66544-0 15894097

[26] Harvey JM Clark GM Osborne CK Allred DC Estrogen receptor status by immunohistochemistry is superior to the ligand-binding assay for predicting response to adjuvant endocrine therapy in breast cancer. J Clin Oncol (1999) 17:1474–81. 10.1200/JCO.1999.17.5.1474 10334533

[27] Bardou VJ Arpino G Elledge RM Osborne CK Clark GM. Progesterone receptor status significantly improves outcome prediction over estrogen receptor status alone for adjuvant endocrine therapy in two large breast cancer databases. J Clin Oncol (2003) 21:1973–9. 10.1200/JCO.2003.09.099 12743151

[28] Dean-Colomb W Esteva FJ. Her2-positive breast cancer: herceptin and beyond. Eur J Cancer (2008) 44:2806–12. 10.1016/j.ejca.2008.09.013 19022660

[29] Cortazar P Zhang L Untch M Mehta K Costantino JP Wolmark N Pathological complete response and long-term clinical benefit in breast cancer: the CTNeoBC pooled analysis. Lancet (2014) 384:164–72. 10.1016/S0140-6736(13)62422-8 24529560

[30] Qi JP Yang YL Zhu H Wang J Jia Y Liu N Expression of the androgen receptor and its correlation with molecular subtypes in 980 Chinese breast cancer patients. Breast Cancer (Auckl) (2012) 6:1–8. 10.4137/BCBCR.S8323 22259247 PMC3256731

[31] Gucalp A Tolaney S Isakoff SJ Ingle JN Liu MC Carey LA Phase II trial of bicalutamide in patients with androgen receptor-positive, estrogen receptor-negative metastatic breast cancer. Clin Cancer Res (2013) 19:5505–12. 10.1158/1078-0432.CCR-12-3327 23965901 PMC4086643

[32] Barton VN D’amato NC Gordon MA Christenson JL Elias A Richer JK. Androgen receptor biology in triple negative breast cancer: a case for classification as AR+ or quadruple negative disease. Horm Cancer (2015) 6:206–13. 10.1007/s12672-015-0232-3 26201402 PMC6897499

[33] Park S Koo JS Kim MS Park HS Lee JS Lee JS Androgen receptor expression is significantly associated with better outcomes in estrogen receptor-positive breast cancers. Ann Oncol (2011) 22:1755–62. 10.1093/annonc/mdq678 21310761

[34] Gucalp A Traina TA. Targeting the androgen receptor in triple-negative breast cancer. Curr Probl Cancer (2016) 40:141–50. 10.1016/j.currproblcancer.2016.09.004 27816190 PMC5580391

[35] Lee E Moon A. Identification of biomarkers for breast cancer using databases. J Cancer Prev (2016) 21:235–42. 10.15430/JCP.2016.21.4.235 28053957 PMC5207607

[36] Sorlie T Perou CM Tibshirani R Aas T Geisler S Johnsen H Gene expression patterns of breast carcinomas distinguish tumor subclasses with clinical implications. Proc Natl Acad Sci U S A (2001) 98:10869–74. 10.1073/pnas.191367098 11553815 PMC58566

[37] Sorlie T Tibshirani R Parker J Hastie T Marron JS Nobel A Repeated observation of breast tumor subtypes in independent gene expression data sets. Proc Natl Acad Sci U S A (2003) 100:8418–23. 10.1073/pnas.0932692100 12829800 PMC166244

[38] Prat A Parker JS Karginova O Fan C Livasy C Herschkowitz JI Phenotypic and molecular characterization of the claudin-low intrinsic subtype of breast cancer. Breast Cancer Res (2010) 12:R68. 10.1186/bcr2635 20813035 PMC3096954

[39] Farmer P Bonnefoi H Becette V Tubiana-Hulin M Fumoleau P Larsimont D Identification of molecular apocrine breast tumours by microarray analysis. Oncogene (2005) 24:4660–71. 10.1038/sj.onc.1208561 15897907

[40] Wirapati P Sotiriou C Kunkel S Farmer P Pradervand S Haibe-Kains B Meta-analysis of gene expression profiles in breast cancer: toward a unified understanding of breast cancer subtyping and prognosis signatures. Breast Cancer Res (2008) 10:R65 10.1186/bcr2124 18662380 PMC2575538

[41] Korde LA Lusa L Mcshane L Lebowitz PF Lukes L Camphausen K Gene expression pathway analysis to predict response to neoadjuvant docetaxel and capecitabine for breast cancer. Breast Cancer Res Treat (2010) 119:685–99. 10.1007/s10549-009-0651-3 20012355 PMC5892182

[42] Early Breast Cancer Trialists’ Collaborative Group Davies C Godwin J Gray R Clarke M Cutter D Relevance of breast cancer hormone receptors and other factors to the efficacy of adjuvant tamoxifen: patient-level meta-analysis of randomised trials. Lancet (2011) 378:771–84. 10.1016/S0140-6736(11)60993-8 21802721 PMC3163848

[43] Cancer Genome Atlas N. Comprehensive molecular portraits of human breast tumours. Nature (2012) 490:61–70. 10.1038/nature11412 23000897 PMC3465532

[44] Curtis C Shah SP Chin SF Turashvili G Rueda OM Dunning MJ The genomic and transcriptomic architecture of 2,000 breast tumours reveals novel subgroups. Nature (2012) 486:346–52. 10.1038/nature10983 22522925 PMC3440846

[45] Ali HR Rueda OM Chin SF Curtis C Dunning MJ Aparicio SA Genome-driven integrated classification of breast cancer validated in over 7,500 samples. Genome Biol (2014) 15:431. 10.1186/s13059-014-0431-1 25164602 PMC4166472

[46] Paik S Shak S Tang G Kim C Baker J Cronin M A multigene assay to predict recurrence of tamoxifen-treated, node-negative breast cancer. N Engl J Med (2004) 351:2817–26. 10.1056/NEJMoa041588 15591335

[47] Dubsky P Brase JC Jakesz R Rudas M Singer CF Greil R The EndoPredict score provides prognostic information on late distant metastases in ER+/HER2- breast cancer patients. Br J Cancer (2013) 109:2959–64. 10.1038/bjc.2013.671 24157828 PMC3859949

[48] Parker JS Mullins M Cheang MC Leung S Voduc D Vickery T Supervised risk predictor of breast cancer based on intrinsic subtypes. J Clin Oncol (2009) 27:1160–7. 10.1200/JCO.2008.18.1370 19204204 PMC2667820

[49] Nielsen TO Parker JS Leung S Voduc D Ebbert M Vickery T A comparison of PAM50 intrinsic subtyping with immunohistochemistry and clinical prognostic factors in tamoxifen-treated estrogen receptor-positive breast cancer. Clin Cancer Res (2010) 16:5222–32. 10.1158/1078-0432.CCR-10-1282 20837693 PMC2970720

[50] Nielsen T Wallden B Schaper C Ferree S Liu S Gao D Analytical validation of the PAM50-based prosigna breast cancer prognostic gene signature assay and nCounter Analysis System using formalin-fixed paraffin-embedded breast tumor specimens. BMC Cancer (2014) 14:177. 10.1186/1471-2407-14-177 24625003 PMC4008304

[51] Sgroi DC Sestak I Cuzick J Zhang Y Schnabel CA Schroeder B Prediction of late distant recurrence in patients with oestrogen-receptor-positive breast cancer: a prospective comparison of the breast-cancer index (BCI) assay, 21-gene recurrence score, and IHC4 in the TransATAC study population. Lancet Oncol (2013) 14:1067–76. 10.1016/S1470-2045(13)70387-5 24035531 PMC3918681

[52] Beca F Polyak K Intratumor heterogeneity in breast cancer. Adv Exp Med Biol (2016) 882:169–89. 10.1007/978-3-319-22909-6_7 26987535

[53] Paik S Tang G Shak S Kim C Baker J Kim W Gene expression and benefit of chemotherapy in women with node-negative, estrogen receptor-positive breast cancer. J Clin Oncol (2006) 24:3726–34. 10.1200/JCO.2005.04.7985 16720680

[54] Partin JF Mamounas EP. Impact of the 21-gene recurrence score assay compared with standard clinicopathologic guidelines in adjuvant therapy selection for node-negative, estrogen receptor-positive breast cancer. Ann Surg Oncol (2011) 18:3399–406. 10.1245/s10434-011-1698-z 21537874

[55] Cheang MC Voduc D Bajdik C Leung S Mckinney S Chia SK Basal-like breast cancer defined by five biomarkers has superior prognostic value than triple-negative phenotype. Clin Cancer Res (2008) 14:1368–76. 10.1158/1078-0432.CCR-07-1658 18316557

[56] Tang P Skinner KA Hicks DG. Molecular classification of breast carcinomas by immunohistochemical analysis: are we ready? Diagn Mol Pathol (2009) 18:125–32. 10.1097/PDM.0b013e31818d107b 19704256

[57] Geyer FC Weigelt B Natrajan R Lambros MB De Biase D Vatcheva R Molecular analysis reveals a genetic basis for the phenotypic diversity of metaplastic breast carcinomas. J Pathol (2010) 220:562–73. 10.1002/path.2675 20099298

[58] Patani N Barbashina V Lambros MB Gauthier A Mansour M Mackay A Direct evidence for concurrent morphological and genetic heterogeneity in an invasive ductal carcinoma of triple-negative phenotype. J Clin Pathol (2011) 64:822–8. 10.1136/jclinpath-2011-200135 21676924

[59] Nik-Zainal S Van Loo P Wedge DC Alexandrov LB Greenman CD Lau KW The life history of 21 breast cancers. Cell (2012) 149:994–1007. 10.1016/j.cell.2012.04.023 22608083 PMC3428864

[60] Balko JM Giltnane JM Wang K Schwarz LJ Young CD Cook RS Molecular profiling of the residual disease of triple-negative breast cancers after neoadjuvant chemotherapy identifies actionable therapeutic targets. Cancer Discov (2014) 4:232–45. 10.1158/2159-8290.CD-13-0286 24356096 PMC3946308

[61] Zardavas D Maetens M Irrthum A Goulioti T Engelen K Fumagalli D The AURORA initiative for metastatic breast cancer. Br J Cancer (2014) 111:1881–7. 10.1038/bjc.2014.341 25225904 PMC4229627

[62] Kuukasjarvi T Karhu R Tanner M Kahkonen M Schaffer A Nupponen N Genetic heterogeneity and clonal evolution underlying development of asynchronous metastasis in human breast cancer. Cancer Res (1997) 57:1597–604. 9108466

[63] Shah SP Morin RD Khattra J Prentice L Pugh T Burleigh A Mutational evolution in a lobular breast tumour profiled at single nucleotide resolution. Nature (2009) 461:809–13. 10.1038/nature08489 19812674

[64] Simpson PT Reis-Filho JS Gale T Lakhani SR. Molecular evolution of breast cancer. J Pathol (2005) 205:248–54. 10.1002/path.1691 15641021

[65] Kalinsky K Heguy A Bhanot UK Patil S Moynahan ME. PIK3CA mutations rarely demonstrate genotypic intratumoral heterogeneity and are selected for in breast cancer progression. Breast Cancer Res Treat (2011) 129:635–43. 10.1007/s10549-011-1601-4 21617917

[66] Ding L Ellis MJ Li S Larson DE Chen K Wallis JW Genome remodelling in a basal-like breast cancer metastasis and xenograft. Nature (2010) 464:999–1005. 10.1038/nature08989 20393555 PMC2872544

[67] Hull DF III Clark GM Osborne CK Chamness GC Knight WA III Mcguire WL. Multiple estrogen receptor assays in human breast cancer. Cancer Res (1983) 43:413–6. 6847780

[68] Amir E Miller N Geddie W Freedman O Kassam F Simmons C Prospective study evaluating the impact of tissue confirmation of metastatic disease in patients with breast cancer. J Clin Oncol (2012) 30:587–92. 10.1200/JCO.2010.33.5232 22124102 PMC5015424

[69] Lindstrom LS Karlsson E Wilking UM Johansson U Hartman J Lidbrink EK Clinically used breast cancer markers such as estrogen receptor, progesterone receptor, and human epidermal growth factor receptor 2 are unstable throughout tumor progression. J Clin Oncol (2012) 30:2601–8. 10.1200/JCO.2011.37.2482 22711854

[70] Allegra JC Barlock A Huff KK Lippman ME. Changes in multiple or sequential estrogen receptor determinations in breast cancer. Cancer (1980) 45:792–4. 10.1002/1097-0142(19800215)45:4<792::AID-CNCR2820450430>3.0.CO;2-X 7357496

[71] Wicha MS. Cancer stem cell heterogeneity in hereditary breast cancer. Breast Cancer Res (2008) 10:105. 10.1186/bcr1990 18423071 PMC2397530

[72] Wu JM Fackler MJ Halushka MK Molavi DW Taylor ME Teo WW Heterogeneity of breast cancer metastases: comparison of therapeutic target expression and promoter methylation between primary tumors and their multifocal metastases. Clin Cancer Res (2008) 14:1938–46. 10.1158/1078-0432.CCR-07-4082 18381931 PMC2965068

[73] Becker TE Ellsworth RE Deyarmin B Patney HL Jordan RM Hooke JA The genomic heritage of lymph node metastases: implications for clinical management of patients with breast cancer. Ann Surg Oncol (2008) 15:1056–63. 10.1245/s10434-008-9815-3 18246400

[74] Almendro V Kim HJ Cheng YK Gonen M Itzkovitz S Argani P Genetic and phenotypic diversity in breast tumor metastases. Cancer Res (2014) 74:1338–48. 10.1158/0008-5472.CAN-13-2357-T 24448237 PMC3963810

[75] Blau CA Ramirez AB Blau S Pritchard CC Dorschner MO Schmechel SC A distributed network for intensive longitudinal monitoring in metastatic triple-negative breast cancer. J Natl Compr Canc Netw (2016) 14:8–17. 10.6004/jnccn.2016.0003 26733551 PMC4970582

[76] Van Poznak C Somerfield MR Bast RC Cristofanilli M Goetz MP Gonzalez-Angulo AM Use of biomarkers to guide decisions on systemic therapy for women with metastatic breast cancer: American Society of Clinical Oncology Clinical Practice Guideline. J Clin Oncol (2015) 33:2695–704. 10.1200/JCO.2015.61.1459 26195705 PMC5478102

[77] Davis BW Zava DT Locher GW Goldhirsch A Hartmann WH. Receptor heterogeneity of human breast cancer as measured by multiple intratumoral assays of estrogen and progesterone receptor. Eur J Cancer Clin Oncol (1984) 20:375–82. 10.1016/0277-5379(84)90084-1 6323188

[78] Nassar A Radhakrishnan A Cabrero IA Cotsonis GA Cohen C. Intratumoral heterogeneity of immunohistochemical marker expression in breast carcinoma: a tissue microarray-based study. Appl Immunohistochem Mol Morphol (2010) 18:433–41. 10.1097/PAI.0b013e3181dddb20 20485156

[79] Pertschuk LP Axiotis CA Feldman JG Kim YD Karavattayhayyil SJ Braithwaite L. Marked intratumoral heterogeneity of the proto-oncogene Her-2/neu determined by three different detection systems. Breast J (1999) 5:369–74. 10.1046/j.1524-4741.1999.97088.x 11348316

[80] Allison KH Dintzis SM Schmidt RA. Frequency of HER2 heterogeneity by fluorescence in situ hybridization according to CAP expert panel recommendations: time for a new look at how to report heterogeneity. Am J Clin Pathol (2011) 136:864–71. 10.1309/AJCPXTZSKBRIP07W 22095371

[81] Seol H Lee HJ Choi Y Lee HE Kim YJ Kim JH Intratumoral heterogeneity of HER2 gene amplification in breast cancer: its clinicopathological significance. Mod Pathol (2012) 25:938–48. 10.1038/modpathol.2012.36 22388760

[82] Glockner S Buurman H Kleeberger W Lehmann U Kreipe H. Marked intratumoral heterogeneity of c-myc and cyclinD1 but not of c-erbB2 amplification in breast cancer. Lab Invest (2002) 82:1419–26. 10.1097/01.LAB.0000032371.16521.40 12379776

[83] Andersson J Linderholm B Bergh J Elmberger G. HER-2/neu (c-erbB-2) evaluation in primary breast carcinoma by fluorescent in situ hybridization and immunohistochemistry with special focus on intratumor heterogeneity and comparison of invasive and in situ components. Appl Immunohistochem Mol Morphol (2004) 12:14–20. 10.1097/00129039-200403000-00003 15163013

[84] Vance GH Barry TS Bloom KJ Fitzgibbons PL Hicks DG Jenkins RB Genetic heterogeneity in HER2 testing in breast cancer: panel summary and guidelines. Arch Pathol Lab Med (2009) 133:611–2. 10.1043/1543-2165-133.4.611 19391661

[85] Chhieng DC Frost AR Niwas S Weiss H Grizzle WE Beeken S Intratumor heterogeneity of biomarker expression in breast carcinomas. Biotech Histochem (2004) 79:25–36. 10.1080/10520290410001715237 15223751

[86] Focke CM Decker T Van Diest PJ. Intratumoral heterogeneity of Ki67 expression in early breast cancers exceeds variability between individual tumours. Histopathology (2016) 69:849–61. 10.1111/his.13007 27270560

[87] Siitonen SM Isola JJ Rantala IS Helin HJ. Intratumor variation in cell proliferation in breast carcinoma as determined by antiproliferating cell nuclear antigen monoclonal antibody and automated image analysis. Am J Clin Pathol (1993) 99:226–31. 10.1093/ajcp/99.3.226 8095363

[88] de Azambuja E Cardoso F De Castro G Jr Colozza M Mano MS Durbecq V Ki-67 as prognostic marker in early breast cancer: a meta-analysis of published studies involving 12,155 patients. Br J Cancer (2007) 96:1504–13. 10.1038/sj.bjc.6603756 17453008 PMC2359936

[89] Yerushalmi R Woods R Ravdin PM Hayes MM Gelmon KA. Ki67 in breast cancer: prognostic and predictive potential. Lancet Oncol (2010) 11:174–83. 10.1016/S1470-2045(09)70262-1 20152769

[90] Ingolf JB Russalina M Simona M Julia R Gilda S Bohle RM Can ki-67 play a role in prediction of breast cancer patients’ response to neoadjuvant chemotherapy? Biomed Res Int (2014) 2014:628217. 10.1155/2014/628217 24783217 PMC3982412

[91] Cserni G Voros A Liepniece-Karele I Bianchi S Vezzosi V Grabau D Distribution pattern of the Ki67 labelling index in breast cancer and its implications for choosing cut-off values. Breast (2014) 23:259–63. 10.1016/j.breast.2014.02.003 24613255

[92] Aleskandarany MA Green AR Ashankyty I Elmouna A Diez-Rodriguez M Nolan CC Impact of intratumoural heterogeneity on the assessment of Ki67 expression in breast cancer. Breast Cancer Res Treat (2016) 158:287–95. 10.1007/s10549-016-3893-x 27380874

[93] Zardavas D Irrthum A Swanton C Piccart M. Clinical management of breast cancer heterogeneity. Nat Rev Clin Oncol (2015) 12:381–94. 10.1038/nrclinonc.2015.73 25895611

[94] Fehm T Sagalowsky A Clifford E Beitsch P Saboorian H Euhus D Cytogenetic evidence that circulating epithelial cells in patients with carcinoma are malignant. Clin Cancer Res (2002) 8:2073–84. 12114406

[95] Allard WJ Matera J Miller MC Repollet M Connelly MC Rao C Tumor cells circulate in the peripheral blood of all major carcinomas but not in healthy subjects or patients with nonmalignant diseases. Clin Cancer Res (2004) 10:6897–904. 10.1158/1078-0432.CCR-04-0378 15501967

[96] Hartkopf AD Wagner P Wallwiener D Fehm T Rothmund R. Changing levels of circulating tumor cells in monitoring chemotherapy response in patients with metastatic breast cancer. Anticancer Res (2011) 31:979–84. 21498725

[97] Aurilio G Sciandivasci A Munzone E Sandri MT Zorzino L Cassatella MC Prognostic value of circulating tumor cells in primary and metastatic breast cancer. Expert Rev Anticancer Ther (2012) 12:203–14. 10.1586/era.11.208 22316368

[98] Hayashi N Nakamura S Tokuda Y Shimoda Y Yagata H Yoshida A Prognostic value of HER2-positive circulating tumor cells in patients with metastatic breast cancer. Int J Clin Oncol (2012) 17:96–104. 10.1007/s10147-011-0260-0 21671160 PMC3860324

[99] Mego M Gao H Lee BN Cohen EN Tin S Giordano A Prognostic value of EMT-circulating tumor cells in metastatic breast cancer patients undergoing high-dose chemotherapy with autologous hematopoietic stem cell transplantation. J Cancer (2012) 3:369–80. 10.7150/jca.5111 23074378 PMC3471078

[100] Pierga JY Hajage D Bachelot T Delaloge S Brain E Campone M High independent prognostic and predictive value of circulating tumor cells compared with serum tumor markers in a large prospective trial in first-line chemotherapy for metastatic breast cancer patients. Ann Oncol (2012) 23:618–24. 10.1093/annonc/mdr263 21642515

[101] Zhao L Li P Li F Yang Y Liu N Cai L. The prognostic value of circulating tumor cells lacking cytokeratins in metastatic breast cancer patients. J Cancer Res Ther (2013) 9:29–37. 10.4103/0973-1482.110353 23575071

[102] Smerage JB Barlow WE Hortobagyi GN Winer EP Leyland-Jones B Srkalovic G Circulating tumor cells and response to chemotherapy in metastatic breast cancer: SWOG S0500. J Clin Oncol (2014) 32:3483–9. 10.1200/JCO.2014.56.2561 24888818 PMC4209100

[103] Janni WJ Rack B Terstappen LW Pierga JY Taran FA Fehm T Pooled analysis of the prognostic relevance of circulating tumor cells in primary breast cancer. Clin Cancer Res (2016) 22:2583–93. 10.1158/1078-0432.CCR-15-1603 26733614

[104] Lv Q Gong L Zhang T Ye J Chai L Ni C Prognostic value of circulating tumor cells in metastatic breast cancer: a systemic review and meta-analysis. Clin Transl Oncol (2016) 18:322–30. 10.1007/s12094-015-1372-1 26260915

[105] Wicha MS Hayes DF Circulating tumor cells: not all detected cells are bad and not all bad cells are detected. J Clin Oncol (2011) 29:1508–11. 10.1200/JCO.2010.34.0026 21422428

[106] Hayes DF Paoletti C. Circulating tumour cells: insights into tumour heterogeneity. J Intern Med (2013) 274:137–43. 10.1111/joim.12047 23844916

[107] Bidard FC Proudhon C Pierga JY Circulating tumor cells in breast cancer. Mol Oncol (2016) 10:418–30. 10.1016/j.molonc.2016.01.001 26809472 PMC5528978

[108] Sieuwerts AM Mostert B Bolt-De Vries J Peeters D De Jongh FE Stouthard JM mRNA and microRNA expression profiles in circulating tumor cells and primary tumors of metastatic breast cancer patients. Clin Cancer Res (2011) 17:3600–18. 10.1158/1078-0432.CCR-11-0255 21505063

[109] Powell AA Talasaz AH Zhang H Coram MA Reddy A Deng G Single cell profiling of circulating tumor cells: transcriptional heterogeneity and diversity from breast cancer cell lines. PLoS One (2012) 7:e33788. 10.1371/journal.pone.0033788 22586443 PMC3346739

[110] Babayan A Hannemann J Spotter J Muller V Pantel K Joosse SA. Heterogeneity of estrogen receptor expression in circulating tumor cells from metastatic breast cancer patients. PLoS One (2013) 8:e75038. 10.1371/journal.pone.0075038 24058649 PMC3776726

[111] Markou A Farkona S Schiza C Efstathiou T Kounelis S Malamos N PIK3CA mutational status in circulating tumor cells can change during disease recurrence or progression in patients with breast cancer. Clin Cancer Res (2014) 20:5823–34. 10.1158/1078-0432.CCR-14-0149 25398847

[112] Gasch C Plummer PN Jovanovic L Mcinnes LM Wescott D Saunders CM Heterogeneity of miR-10b expression in circulating tumor cells. Sci Rep (2015) 5:15980. 10.1038/srep15980 26522916 PMC4629160

[113] Pestrin M Salvianti F Galardi F De Luca F Turner N Malorni L Heterogeneity of PIK3CA mutational status at the single cell level in circulating tumor cells from metastatic breast cancer patients. Mol Oncol (2015) 9:749–57. 10.1016/j.molonc.2014.12.001 25539732 PMC5528771

[114] De Luca F Rotunno G Salvianti F Galardi F Pestrin M Gabellini S Mutational analysis of single circulating tumor cells by next generation sequencing in metastatic breast cancer. Oncotarget (2016) 7:26107–19. 10.18632/oncotarget.8431 27034166 PMC5041968

[115] Grover PK Cummins AG Price TJ Roberts-Thomson IC Hardingham JE. Circulating tumour cells: the evolving concept and the inadequacy of their enrichment by EpCAM-based methodology for basic and clinical cancer research. Ann Oncol (2014) 25:1506–16. 10.1093/annonc/mdu018 24651410

[116] Hyun KA Koo GB Han H Sohn J Choi W Kim SI Epithelial-to-mesenchymal transition leads to loss of EpCAM and different physical properties in circulating tumor cells from metastatic breast cancer. Oncotarget (2016) 7:24677–87. 10.18632/oncotarget.8250 27013581 PMC5029733

[117] Bulfoni M Gerratana L Del Ben F Marzinotto S Sorrentino M Turetta M In patients with metastatic breast cancer the identification of circulating tumor cells in epithelial-to-mesenchymal transition is associated with a poor prognosis. Breast Cancer Res (2016) 18:30. 10.1186/s13058-016-0687-3 26961140 PMC4784394

[118] Hayes DF Walker TM Singh B Vitetta ES Uhr JW Gross S Monitoring expression of HER-2 on circulating epithelial cells in patients with advanced breast cancer. Int J Oncol (2002) 21:1111–7. 12370762 10.3892/ijo.21.5.1111

[119] Georgoulias V Bozionelou V Agelaki S Perraki M Apostolaki S Kallergi G Trastuzumab decreases the incidence of clinical relapses in patients with early breast cancer presenting chemotherapy-resistant CK-19mRNA-positive circulating tumor cells: results of a randomized phase II study. Ann Oncol (2012) 23:1744–50. 10.1093/annonc/mds020 22377561

[120] Mikulova V Cabinakova M Janatkova I Mestek O Zima T Tesarova P. Detection of circulating tumor cells during follow-up of patients with early breast cancer: clinical utility for monitoring of therapy efficacy. Scand J Clin Lab Invest (2014) 74:132–42. 10.3109/00365513.2013.864784 24350991

[121] Agelaki S Kalykaki A Markomanolaki H Papadaki MA Kallergi G Hatzidaki D Efficacy of lapatinib in therapy-resistant HER2-positive circulating tumor cells in metastatic breast cancer. PLoS One (2015) 10:e0123683. 10.1371/journal.pone.0123683 26083256 PMC4471111

[122] Bidard FC Fehm T Ignatiadis M Smerage JB Alix-Panabieres C Janni W Clinical application of circulating tumor cells in breast cancer: overview of the current interventional trials. Cancer Metastasis Rev (2013) 32:179–88. 10.1007/s10555-012-9398-0 23129208 PMC3655223

[123] Schramm A Friedl TW Schochter F Scholz C De Gregorio N Huober J Therapeutic intervention based on circulating tumor cell phenotype in metastatic breast cancer: concept of the DETECT study program. Arch Gynecol Obstet (2016) 293:271–81. 10.1007/s00404-015-3879-7 26354331

[124] Fiegl M Tueni C Schenk T Jakesz R Gnant M Reiner A Interphase cytogenetics reveals a high incidence of aneuploidy and intra-tumour heterogeneity in breast cancer. Br J Cancer (1995) 72:51–5. 10.1038/bjc.1995.276 7599066 PMC2034116

[125] Torres L Ribeiro FR Pandis N Andersen JA Heim S Teixeira MR. Intratumor genomic heterogeneity in breast cancer with clonal divergence between primary carcinomas and lymph node metastases. Breast Cancer Res Treat (2007) 102:143–55. 10.1007/s10549-006-9317-6 16906480

[126] Banerji S Cibulskis K Rangel-Escareno C Brown KK Carter SL Frederick AM Sequence analysis of mutations and translocations across breast cancer subtypes. Nature (2012) 486:405–9. 10.1038/nature11154 22722202 PMC4148686

[127] Ellis MJ Ding L Shen D Luo J Suman VJ Wallis JW Whole-genome analysis informs breast cancer response to aromatase inhibition. Nature (2012) 486:353–60. 10.1038/nature11143 22722193 PMC3383766

[128] Shah SP Roth A Goya R Oloumi A Ha G Zhao Y The clonal and mutational evolution spectrum of primary triple-negative breast cancers. Nature (2012) 486:395–9. 10.1038/nature10933 22495314 PMC3863681

[129] Stephens PJ Tarpey PS Davies H Van Loo P Greenman C Wedge DC The landscape of cancer genes and mutational processes in breast cancer. Nature (2012) 486:400–4. 10.1038/nature11017 22722201 PMC3428862

[130] Ellsworth RE Toro AL Blackburn HL Decewicz A Deyarmin B Mamula KA Molecular heterogeneity in primary breast carcinomas and axillary lymph node metastases assessed by genomic fingerprinting analysis. Cancer Growth Metastasis (2015) 8:15–24. 10.4137/CGM.S29490 26279627 PMC4511091

[131] Navin N Krasnitz A Rodgers L Cook K Meth J Kendall J Inferring tumor progression from genomic heterogeneity. Genome Res (2010) 20:68–80. 10.1101/gr.099622.109 19903760 PMC2798832

[132] Martelotto LG Ng CK Piscuoglio S Weigelt B Reis-Filho JS. Breast cancer intra-tumor heterogeneity. Breast Cancer Res (2014) 16:210. 10.1186/bcr3658 25928070 PMC4053234

[133] Juric D Castel P Griffith M Griffith OL Won HH Ellis H Convergent loss of PTEN leads to clinical resistance to a PI(3)Kalpha inhibitor. Nature (2015) 518:240–4. 10.1038/nature13948 25409150 PMC4326538

[134] Marusyk A Almendro V Polyak K. Intra-tumour heterogeneity: a looking glass for cancer? Nat Rev Cancer (2012) 12:323–34. 10.1038/nrc3261 22513401

[135] Almendro V Marusyk A Polyak K. Cellular heterogeneity and molecular evolution in cancer. Annu Rev Pathol (2013) 8:277–302. 10.1146/annurev-pathol-020712-163923 23092187

[136] Reynolds PA Sigaroudinia M Zardo G Wilson MB Benton GM Miller CJ Tumor suppressor p16INK4A regulates polycomb-mediated DNA hypermethylation in human mammary epithelial cells. J Biol Chem (2006) 281:24790–802. 10.1074/jbc.M604175200 16766534

[137] Pasquali L Bedeir A Ringquist S Styche A Bhargava R Trucco G. Quantification of CpG island methylation in progressive breast lesions from normal to invasive carcinoma. Cancer Lett (2007) 257:136–44. 10.1016/j.canlet.2007.07.010 17706863

[138] Sunami E Shinozaki M Sim MS Nguyen SL Vu AT Giuliano AE Estrogen receptor and HER2/neu status affect epigenetic differences of tumor-related genes in primary breast tumors. Breast Cancer Res (2008) 10:R46. 10.1186/bcr2098 18485221 PMC2481494

[139] Corre G Stockholm D Arnaud O Kaneko G Vinuelas J Yamagata Y Stochastic fluctuations and distributed control of gene expression impact cellular memory. PLoS One (2014) 9:e115574. 10.1371/journal.pone.0115574 25531401 PMC4274012

[140] Kendrick H Regan JL Magnay FA Grigoriadis A Mitsopoulos C Zvelebil M Transcriptome analysis of mammary epithelial subpopulations identifies novel determinants of lineage commitment and cell fate. BMC Genomics (2008) 9:591. 10.1186/1471-2164-9-591 19063729 PMC2629782

[141] Koren S Bentires-Alj M. Breast tumor heterogeneity: source of fitness, hurdle for therapy. Mol Cell (2015) 60:537–46. 10.1016/j.molcel.2015.10.031 26590713

[142] Ince TA Richardson AL Bell GW Saitoh M Godar S Karnoub AE Transformation of different human breast epithelial cell types leads to distinct tumor phenotypes. Cancer Cell (2007) 12:160–70. 10.1016/j.ccr.2007.06.013 17692807

[143] Van Keymeulen A Rocha AS Ousset M Beck B Bouvencourt G Rock J Distinct stem cells contribute to mammary gland development and maintenance. Nature (2011) 479:189–93. 10.1038/nature10573 21983963

[144] Keller PJ Arendt LM Skibinski A Logvinenko T Klebba I Dong S Defining the cellular precursors to human breast cancer. Proc Natl Acad Sci U S A (2012) 109:2772–7. 10.1073/pnas.1017626108 21940501 PMC3286919

[145] Melchor L Molyneux G Mackay A Magnay FA Atienza M Kendrick H Identification of cellular and genetic drivers of breast cancer heterogeneity in genetically engineered mouse tumour models. J Pathol (2014) 233:124–37. 10.1002/path.4345 24615332

[146] Tao L Van Bragt MP Li Z. A long-lived luminal subpopulation enriched with alveolar progenitors serves as cellular origin of heterogeneous mammary tumors. Stem Cell Reports (2015) 5:60–74. 10.1016/j.stemcr.2015.05.014 26120057 PMC4618443

[147] Koren S Reavie L Couto JP De Silva D Stadler MB Roloff T PIK3CA(H1047R) induces multipotency and multi-lineage mammary tumours. Nature (2015) 525:114–8. 10.1038/nature14669 26266975

[148] Gupta PB Fillmore CM Jiang G Shapira SD Tao K Kuperwasser C Stochastic state transitions give rise to phenotypic equilibrium in populations of cancer cells. Cell (2011) 146:633–44. 10.1016/j.cell.2011.07.026 21854987

[149] Kreso A Dick JE Evolution of the cancer stem cell model. Cell Stem Cell (2014) 14:275–91. 10.1016/j.stem.2014.02.006 24607403

[150] Chaffer CL Brueckmann I Scheel C Kaestli AJ Wiggins PA Rodrigues LO Normal and neoplastic nonstem cells can spontaneously convert to a stem-like state. Proc Natl Acad Sci U S A (2011) 108:7950–5. 10.1073/pnas.1102454108 21498687 PMC3093533

[151] Skibinski A Kuperwasser C. The origin of breast tumor heterogeneity. Oncogene (2015) 34:5309–16. 10.1038/onc.2014.475 25703331 PMC4734640

[152] Van Keymeulen A Lee MY Ousset M Brohee S Rorive S Giraddi RR Reactivation of multipotency by oncogenic PIK3CA induces breast tumour heterogeneity. Nature (2015) 525:119–23. 10.1038/nature14665 26266985

[153] Nowell PC. The clonal evolution of tumor cell populations. Science (1976) 194:23–8. 10.1126/science.959840 959840

[154] Bissell MJ Hines WC. Why don’t we get more cancer? A proposed role of the microenvironment in restraining cancer progression. Nat Med (2011) 17:320–9. 10.1038/nm.2328 21383745 PMC3569482

[155] Quail DF Joyce JA. Microenvironmental regulation of tumor progression and metastasis. Nat Med (2013) 19:1423–37. 10.1038/nm.3394 24202395 PMC3954707

[156] Ghajar CM Peinado H Mori H Matei IR Evason KJ Brazier H The perivascular niche regulates breast tumour dormancy. Nat Cell Biol (2013) 15:807–17. 10.1038/ncb2767 23728425 PMC3826912

[157] Ng CK Pemberton HN Reis-Filho JS. Breast cancer intratumor genetic heterogeneity: causes and implications. Expert Rev Anticancer Ther (2012) 12:1021–32. 10.1586/era.12.85 23030222

[158] Aparicio S Caldas C The implications of clonal genome evolution for cancer medicine. N Engl J Med (2013) 368:842–51. 10.1056/NEJMra1204892 23445095

[159] Bedard PL Hansen AR Ratain MJ Siu LL Tumour heterogeneity in the clinic. Nature (2013) 501:355–64. 10.1038/nature12627 24048068 PMC5224525

